# In Vivo Endoscopic Optical Coherence Tomography of the Healthy Human Oral Mucosa: Qualitative and Quantitative Image Analysis

**DOI:** 10.3390/diagnostics10100827

**Published:** 2020-10-15

**Authors:** Marius Albrecht, Christian Schnabel, Juliane Mueller, Jonas Golde, Edmund Koch, Julia Walther

**Affiliations:** 1 Department of Medical Physics and Biomedical Engineering, Technische Universitaet Dresden, Carl Gustav Carus Faculty of Medicine, Fetscherstraße 74, 01307 Dresden, Germany; marius.albrecht@mailbox.tu-dresden.de (M.A.); christian.schnabel@tu-dresden.de (C.S.); 2 Department of Anesthesiology and Intensive Care Medicine, Technische Universität Dresden, Clinical Sensoring and Monitoring, Carl Gustav Carus Faculty of Medicine, Fetscherstraße 74, 01307 Dresden, Germany; juliane.mueller3@tu-dresden.de (J.M.); jonas.golde@tu-dresden.de (J.G.); edmund.koch@tu-dresden.de (E.K.)

**Keywords:** optical coherence tomography, endoscopy, noninvasive, healthy human oral mucosa, epithelial thickness, adults, histology

## Abstract

To date, there is still a lack of reliable imaging modalities to improve the quality of consultation, diagnostic and medical examinations of the oral mucosa in dentistry. Even though, optical technologies have become an important element for the detection and treatment of different diseases of soft tissue, for the case of oral screenings the evidence of the benefit in comparison to conventional histopathology is mostly still pending. One promising optical technology for oral diagnostics is optical coherence tomography (OCT). To prove the potential of OCT, even the amount of freely accessible OCT data is not sufficient to describe the variance of healthy human oral soft tissue in vivo. In order to remedy this deficiency, the present study provides in vivo OCT cross sections of the human oral mucosa of the anterior and posterior oral cavity as well as the oropharynx of 47 adult volunteers. A collection of representative OCT cross sections forms the basis for a randomized blinded image analysis by means of seven criteria to assess the main features of the superficial layers of the human oral mucosa and to determine its correlation to regional features known from hematoxylin and eosin (HE) stained histology.

## 1. Introduction

According to the results of the global burden of disease study, diagnosis and treatment of oral cancer is still challenging because of at least >350,000 new cases and >170,000 deaths every year [1]. The most common type of oral cancer is the squamous cell carcinoma (SCC) (90% of all oral malignancies [2]) developing within the few ten microns thin epithelium, which is replaced by a neoplastic tissue associated with a progressive dysplasia [2,3]. The highest incidence is found between the ages of 55 and 75 years corresponding to a mean age of onset of about 63 years for male and 66 years for female patients [4]. With regard to demographic aging, the global incidence of oral cancer is predicted to increase of almost two thirds in 2035 [4]. Even though, treatment methods have been improved over the last decades and leading risk factors like smoking, excessive consumption of alcohol and HPV infection are well-known, the overall 5-year survival remains low of about 63% being significantly decreased in advanced stages [2,3,4,5,6]. The reason for this lies primarily in the high number of initial diagnoses at a late stage underlining the necessity for an improved early diagnosis [4].

With regard to the routine diagnostic process, the general anamnesis is complemented by a visual examination and a subsequent endoscopy for difficult-to-access regions of the posterior oral cavity as well as oropharynx [7]. Depending on the reported symptoms, that may include oral bleeding, persisting pain or progressive swelling, as well as existing visually suspect lesions, the indication for an invasive biopsy with histopathological evaluation is set by means of the medical opinion [7,8]. Although, incisional and excisional biopsies represent the current gold standard of an accurate and complete diagnosis [7,8,9], the procedure is painful and requires a surgical intervention leading to a reduced compliance of the patient [9,10]. Especially for the case of extensive and/or numerous lesions, the decision on the location and size of the tissue sample for a representative biopsy is challenging [9,10]. Moreover, missing out on recurrences and relapses (second primary cancer) or redundant biopsies is problematic [11].

In this context, the implementation of optical techniques provides the opportunity to examine the oral mucosa in a non-invasive and non-ionising way before the decision of starting the tissue extraction [12]. Optical coherence tomography (OCT) is such an emerging optical technology enabling cross-sectional sub-surface in vivo imaging of biological tissues with a common spatial resolution of about 5–20 μm [13,14]. By the implementation of adapted rigid or fiber optics, endoscopic OCT can be used for the non-invasive examination of human oral soft tissue [15,16]. As tissue elements of the mucosal sublayers result in various scattering behaviour, information about the appearance and geometry of the epithelium and the underlying lamina propria becomes accessible [14,17]. Ongoing research has occasionally proposed perspective measurement parameters, such as the mean epithelial thickness, the occurrence of a keratinized layer and the course of the basement membrane, for the detection of (pre-)malignant changes of the oral epithelium by means of OCT [14,17]. Furthermore, OCT image scoring was performed using hamster cheek pouches, concentrating on the presence of irregular epithelial stratification in combination with broadened rete pegs and basal hyperplasia [18]. Beyond that, diagnostic scoring was done on human oral soft tissue in the context of preliminary studies [19]. Moreover, several ex vivo studies as well as an increasing amount of in vivo investigations have described key features of suspicious lesions and oral squamous cell carcinoma, such as the increase in epithelial thickness as well as the loss of the basement membrane during the progress of malignant transformation [17,20]. Regarding oral lesions alongside the upper aerodigestive tract, measurement of epithelial thickness provided large differences resulting in a moderate sensitivity and specificity in order to distinguish noninvasive from invasive proliferation [21]. Along with the process of epithelial thickening, changes in the standard deviation of the OCT signal have been reported offering the possibility to differentiate between the stages of oral dysplasia in a more reliably way [22,23,24]. However, the correct identification of early dysplasia by means of intensity-based OCT was stated to be difficult because of a similar appearance compared to benign lesions [20].

As requirement for the accurate evaluation of pathological changes of the human oral mucosa, healthy oral soft tissue was investigated by only a few studies mostly realized with small numbers of volunteers [25,26,27]. The majority of OCT images were obtained from examinations within the anterior parts of the oral cavity, like the labial, buccal and sublingual mucosa, whereas OCT cross sections of the posterior areas, such as the oropharynx and the palatine tonsils, likewise typical sites of oral cancer, have only been presented rarely. Ridgway et al. [26] showed a collection of OCT cross sections depicting the normal oral mucosa of fifteen different locations as well as a combination of benign and malign lesions. Image material was gained from measurements with a flexible handheld OCT probe including a total of forty-one patients. Within the examples, epithelium was described as hyporeflective layer followed by the basement membrane and the hyperreflective lamina propria consisting of connective tissue, minor salivary glands and blood vessels [26]. Prestin et al. [25] intended to determine reference values for the mean epithelial thickness by investigating 143 healthy test persons. A commercial fiber-based OCT device was used to conduct in vivo measurements at seven points, among them the buccal mucosa, the mouth floor, the palatine arch, the uvula and the hard palate. Further results were published by Stasio et al. [27] involving 28 healthy adult volunteers and six defined areas of the oral soft tissue, the labial mucosa, the buccal mucosa, the gingiva, the mouth floor as well as the ventral and dorsal tongue. Measurements were done by means of a commercial swept-source OCT system with a grip type probe.

Against this background, the demand for a comprehensive study with the aim of assessing the variance of appearance of the healthy oral mucosa, in dependence of the localization within the oral cavity, and with this the ground truth arises [17,26]. Moreover, systematic reviews suggest that the visual interpretation of OCT data sets is often strongly user-dependent due to a lack of established standards for the image analysis [17], which leads to the aim of defining qualitative and quantitative criteria for an improved optical biopsy by OCT. On the other hand, there are several studies dealing with an automatic evaluation of OCT images of oral pathologies [28,29]. Here, changes in the speckle pattern of the epithelium as well as in the signal intensity within the single A-scans offer the possibility to serve as indicators for the development of oral malignancy [28]. Besides, the measurement of the epithelial thickness and the determination of the basement membrane can be done by means of an automatic segmentation algorithm [29]. For this purpose, corresponding histopathological images are used to confirm the findings in the OCT cross sections reaching a solid base for software training in regards to the detection of malign lesions. However, in order to improve the current state of knowledge concerning healthy oral soft tissue, the aim of the presented study is the detection and analysis of in vivo OCT data sets representing characteristic and cancer-relevant parts of normal mucosa alongside the whole oral cavity, which forms the fundamental basis for further developments in functional and endoscopic OCT [26,27,30,31,32,33] as well as subsequent clinical research.

## 2. Materials and Methods

### 2.1. Study Population

Forty-seven healthy adult volunteers participated in the presented in vivo study. Before starting the measurement, volunteers were screened visually by an experienced physician for exclusion criteria including suspicious oral lesions (i.e., erythroplakia, leukoplakia and ulcers), chronic or recently occurred disorders (i.e., mucous membrane and connective tissue diseases) as well as alcohol- and smoking-induced tissue damage. In addition, common oral symptoms (i.e., dry mouth, recurrent pain, gingival bleeding, increased sensitivity) were registered within anamnesis. Reactive changes in the mucosa related to alcohol and tobacco were considered by quantifying alcohol intake (g/day) and smoking habits (cigarettes per day; pack years). According to a systematic review of Burger et al. [34], mucosa was classified as healthy, if the mean consumption did not exceed the tolerable upper alcohol intake level (TUAL; 10–12 g/day for healthy adult women; 20–24 g/day for healthy adult men). Concerning the use of tobacco, the tolerance level corresponds to a frequency of less than 10 cigarettes per day and a total amount of less than 10 pack years leading to a similar likelihood ratio for oral cancer compared to never smokers [35]. Finally, sex and age were noted and used to categorize groups (male/female; <25 years, 25–45 years and >45 years). All participants have declared their informed consent before the examination. The study was approved by the ethics commission of the Faculty of Medicine of the Technische Universitaet Dresden (EK 96032018; 2018-11-16).

### 2.2. Endoscopic OCT System

Acquisition of OCT image series was realized by the use of a customized optical probe (lateral resolution: 17.4 μm, working distance: 7.5 mm) based on a commercial rigid endoscope (8711 AGA, Karl Storz GmbH & Co. KG, length: 200 mm, insertion diameter: 10 mm, angle of view: 0${}^{\circ }$) [36] in combination with a self-built spectrometer-based OCT system using a fiber-coupled superluminescent diode (SLD-371-HP1, Superlumdiodes Ltd., center wavelength: λ = 840 nm, FWHM: λ = 45 nm) and a customized spectrometer [37], providing an axial resolution in air of 11.6 μm. Cross-sectional OCT images (64 B-scans) consisting of 480 A-scans were detected with increments of δx = 5 μm (fast scanning axis) and δy = 10 μm (slow scanning axis) to cover a scanning field of 2.4 mm × 0.6 mm in about 2.5 s (f_A-scan_ = 11.88 kHz). The whole endoscopic OCT system was in accordance with the requirements for a positive ethics committee vote concerning the aspect of disinfection and sterilization.

### 2.3. Measurement Protocol

**Figure 1:**
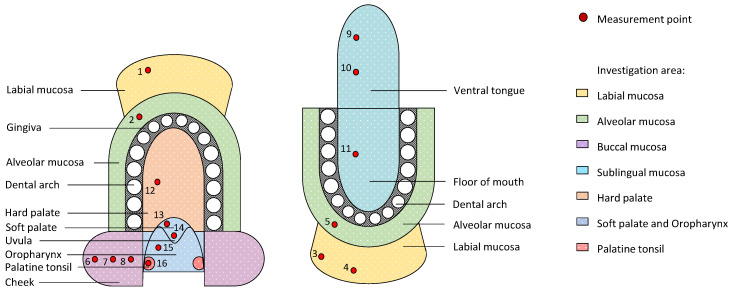
Schematic illustration of the human oral cavity with seven investigation areas (colored zones) and selected measurement points (red dots) in consideration of common sites of oral cancer.

Endoscopic OCT image series were detected at 16 measurement points within seven regions of the oral cavity in accordance to the measurement plan in Figure 1. Corresponding zones that are expected to be characterized by comparable structure and ratio of the mucosal layers are presented in the same color. The sequence of measurements was set from outer to inner oral cavity with additional points alongside the buccal and sublingual region enabling the comparison of anterior and posterior regions. Within the oral vestibule, measurement points were situated alongside the upper and lower part of the labial and alveolar mucosa. In this context the term “alveolar mucosa” defined the oral soft tissue that is located between the attached gingiva and the adjacent labial mucosa. In this study, lining oral mucosa was mainly recorded since deeper structures of the masticatory and specialized oral mucosa, such as strongly keratinized attached gingiva and dorsal tongue, are unreliably detected because of the limited penetration depth as a result of increased backscattering of the probing light. Thus, with the exception of the masticatory mucosa of the hard palate, the presented study particularly considers the measurement of the lining mucosa of the oral cavity and the oro-pharyngeal isthmus with respect to common sites of oral cancer.

The examination with the rigid handheld OCT endoscope was supported by a chin and head rest that ensures the reduction of patient movement and consequently motion artefacts. The entire examination was performed in sitting position whereby measurement points were scanned without direct contact to the local mucosa as well as unilateral on the subject’s right side to minimize measurement time. Recording two stacks of 64 B-scans in each case results in a total examination time of approximately 20 min. To reach all of the points to be investigated, common tools (i.e., tongue depressor, cotton cloth) were used including the minor active involvement of the volunteer (e.g., participants were instructed to turn down their lips for imaging the labial region).

The image processing contained the visual selection of a series of five B-scans for each measurement position being used for the subsequent image analysis. By means of defined criteria (see Section 2.4), each cross section was examined by a prospective physician well-versed in OCT in agreement with a skilled histologist. Quantitative and qualitative evaluation of the selected OCT cross sections has been done with ImageJ [38]. A random choice of 200 images served as training set before the analysis. Finally, a characterization of the anatomical regions by means of OCT was created, summarizing the results of corresponding measurement points and regions.

### 2.4. OCT Image Analysis

**Figure 2:**
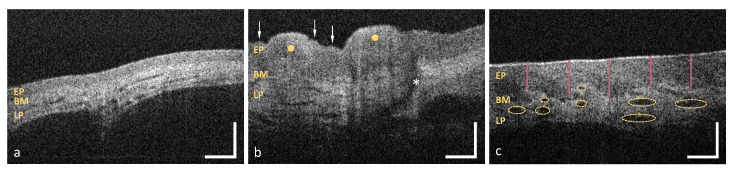
Representative OCT images of the sublingual mucosa (**a**), the hard palate (**b**) and the labial mucosa (**c**) demonstrating the principle of the visual image analysis. EP: epithelium, BM: basement membrane, LP: lamina propria; Arrows in (**b**): epithelial surface alteration, Yellow dots: palatal ridges (uneven surface profile), Asterisk: salivary gland opening; Vertical lines in (**c**): epithelial thickness measurement, Ellipsoidal shapes: marked regions of vascularization. Scale bar: 200 μm.

As illustrated in Table 1, the OCT image analysis was manually performed using a set of criteria concerning the epithelium (EP) and the adjacent lamina propria (LP) (Figure 2). Within this section, evaluation is methodically explained and exemplified by five sample images.

First of all, the integrity of the epithelial surface is assessed by searching for lesions. The surface is called “intact”, if the number of single lesions does not exceed one; otherwise the term “with alterations” is used. The sublingual mucosa depicted in Figure 2a shows an intact surface, whereas the mucosa of the hard palate in Figure 2b contains numerous epithelial surface alterations (arrows). Moreover, the surface profile is evaluated by differentiating between “even” and “uneven” course. The term “even” underlined that the epithelial surface is free from crypts (concave) and ridges (convex) larger than 200 μm in diameter (cp. Figure 2a). Therefore, epithelium with an uneven surface is exemplarily presented by means of the plicae ridges of the hard palate (yellow dots in Figure 2b).

Additionally, the deep structure of the epithelium is categorized into “homogeneous” and “inhomogeneous” by regarding the local tissue reflectivity within a single B-scan. According to former studies that presented healthy oral mucosa [25,26,27], epithelium is expected to be less scattering than the highly reflecting collagen network within the lamina propria. If hypo- or hyperreflective areas are not identified, the epithelium is classified as homogeneous as shown in Figure 2a. In comparison, the masticatory mucosa of the hard palate in Figure 2b shows an inhomogeneous epithelium containing a hyperreflective surface of the ridges (yellow dots in Figure 2b).

Since OCT is an interferometric technique allowing the depth-resolved distance measurement, the optical path length (OPL) of the epithelium (EP, from the surface to the basement membrane BM, Figure 2) is measured at five evenly distributed positions throughout each cross section (red bars in Figure 2c). For this purpose, the OCT cross section was visualized with ImageJ using a 23”-screen. By drawing a vertical line with the straight line tool of Image J, the distance from the top of the epithelial surface to the visually undermost pixel of the hypointense epithelial layer was measured. Here, the position of the basement membrane was determined by eye. Considering the vertical length of the pixel of l = 5 μm and the refractive index of the epithelium of n = 1.37 [25,39] at a center wavelength of λ = 840 nm, the corresponding mean value of the geometrical length is determined for each B-scan. Combining the results of each specific measurement point, sex- and age-specific reference values of healthy oral mucosa are obtained within the investigated regions (see Appendix A).

Furthermore, the basement membrane (BM), defined as junction between the epithelium (EP) and the underlying lamina propria (LP), is visually verified for integrity. The class “intact” is used to characterize a continuous basement membrane without any gaps (cp. Figure 2a). A reduced demarcation of the border is described as “unsharp”. The term “not assessable” is used for the case that the basement membrane could not be depicted due to the reduced penetration depth. Consequently, the evaluation of the lamina propria and the measurement of the epithelial thickness is not possible.

Corresponding to the approach of OCT angiography, the grade of tissue vascularization is estimated by measuring the luminal area of larger vessels and relating the result to the avascular epithelial layer (also referred to as mean vessel density) [40,41]. For this, single or multiple yellow-colored elliptic shapes mark regions of vascularization, as shown in Figure 2c. The sum of the segmented areas is divided by the total area of the detected epithelium determined by a polygonal shape. Depending on the ratio, the vascular supply by major vessels is classified as “low” (<0.10), “moderate“ (0.10–0.30) or “high” (>0.30).

**Figure 3:**
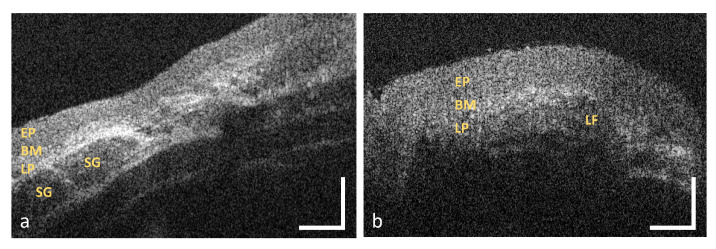
Exemplary OCT images of the oropharynx (**a**) and the palatine tonsil (**b**). The connective tissue of the oropharynx contains minor salivary glands whereas the palatine tonsil includes multiple lymphoid follicles. EP: epithelium, BM: basement membrane, LP: lamina propria, SG: salivary gland, LF: lymphoid follicle. Scale bar: 200 μm.

Additional characteristic features of the lamina propria, such as minor salivary glands (SG in Figure 3a) or lymphoid follicles (LF in Figure 3b), are considered in the last criterion. Salivary glands, which consist of a few lobules and an excretory duct (cp. asterisk in Figure 2b), emerge as round hyporeflective elements [42], embedded within the hyperreflective connective tissue. Lymphoid follicles appear hyporeflective with an inner germinal centre [43].

### 2.5. Statistical Analysis

In order to compare the investigation areas (Figure 1), the percentage distribution of the results of the qualitative evaluation criteria (Table 1) is determined for each measurement point. Concerning the epithelial thickness, a mean value is calculated for each series of five B-scans representing the individual epithelial thickness of the volunteer at the corresponding measurement point. Subsequently, these individual values are used to determine the mean value and standard deviation for the final determination of the epithelial thickness at each measurement point (see Appendix A, Table A1). A similar approach is done for the sex- and age-related groups with the objective to generate an initial data set of reference values. The results for each measurement point and every investigation area are summarized in box plots. The grade of tissue vascularization is analyzed in the same way by calculating mean values for the series of five B-scans of each volunteer and presenting them in box plots depending on the location within the oral cavity.

Table 2 summarized details about the study population and the number of OCT cross sections. Altogether, 3560 OCT images of 47 healthy adult volunteers (N_male_ = 25 and N_female_ = 22) form the basis for the described analysis. The whole sample consisted of fair-skinned participants without oral pigmentation in the visual examination. The mean age corresponds to 29.4 years (range: 20–56 years). Concerning the age groups, 30 subjects (63.8%) were assigned to the mean one (25–45 years). None of the volunteers showed suspicious lesions in the visual examination or described oral symptoms within the anamnesis. Moreover, there was no report of alcohol intake or smoking above the tolerance levels.

The measurement of the anterior oral cavity was feasible in all cases resulting in 235 OCT images for each measurement point (MP1-MP12). The soft palate (MP13) as well as the uvula (MP14) have been accessible in 45 (95.7%) and 40 (85.1%) volunteers, respectively. In addition, the oropharynx (MP15) and the palatine tonsil (MP16) have been able to detect in 29 (61.7%) and 34 (72.3%) volunteers. In 38.4% of the cases, the first of two image stacks (à 64 B-scans) provided visually appropriate B-scans for the OCT image analysis. Using OCT images out of the second stack was especially required for the analysis of the labial and alveolar mucosa due to the flexible soft tissue alongside these regions.

### 2.6. Histological Analysis

Histological correlation was implemented by means of hematoxylin and eosin (HE) stained cross sections [44] of human oral soft tissue, which have been provided by the Institute of Anatomy, Faculty of Medicine, Technische Universitaet Dresden, Germany. Consequently, histological images have not been collected from subjects recruited to the study. A total of seventy histological cross sections, including ten for each investigation area, have been used for the juxtaposition of OCT and histology. Exemplary slides were integrated within a collection of OCT cross sections for characteristic oral sites.

## 3. Results

**Figure 4:**
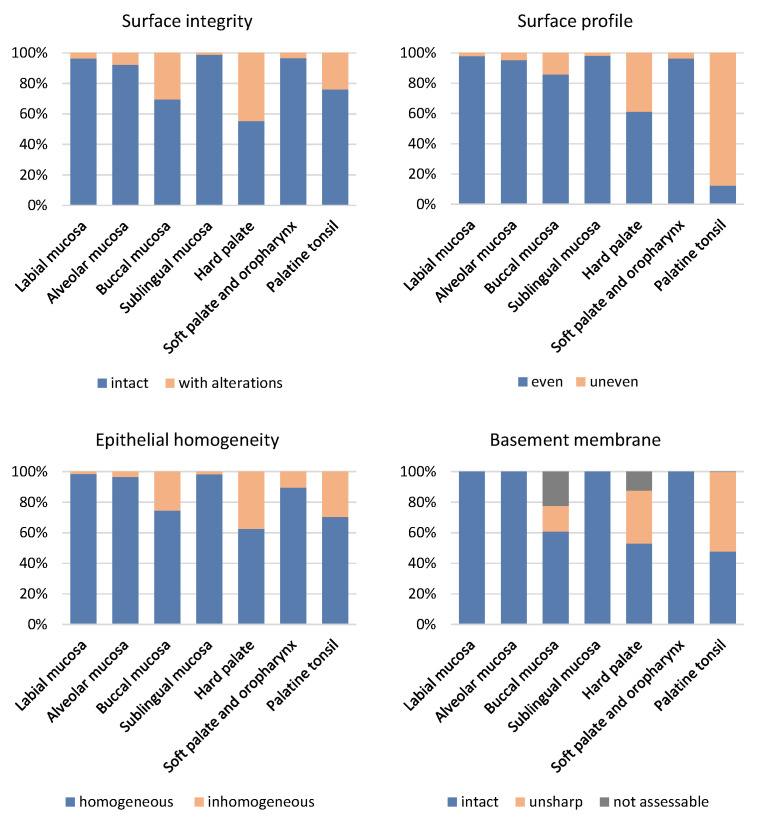
Results of the evaluation of the OCT cross sections within seven oral investigation areas. The image analysis contained the assessment of surface integrity, surface profile, epithelial homogeneity and basement membrane. Percentage values of the qualitative evaluation of the different criteria are presented by the colored bars.

In the following subsections, the results of the OCT image analysis of the defined investigation areas are described in detail based on the evaluation criteria presented in Section 2.4. A summary of the results concerning the qualitative criteria is depicted in the percentage bar chart in Figure 4.

**Figure 5:**
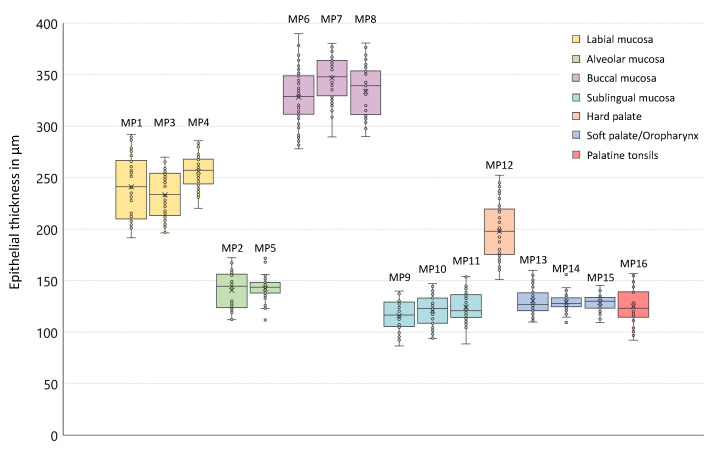
Box plots of the epithelial thickness (age- and gender-independent). The results for all measurement points are presented by colored boxes in accordance with the corresponding investigation areas (cp. Figure 1). The sample median is depicted as central line inside the boxes, the sample mean value corresponds to the cross. The individual mean values are illustrated by circles. The width of the box is equivalent to the interquartile range. Maximum and minimum of the sample are shown by the whiskers.

**Figure 6:**
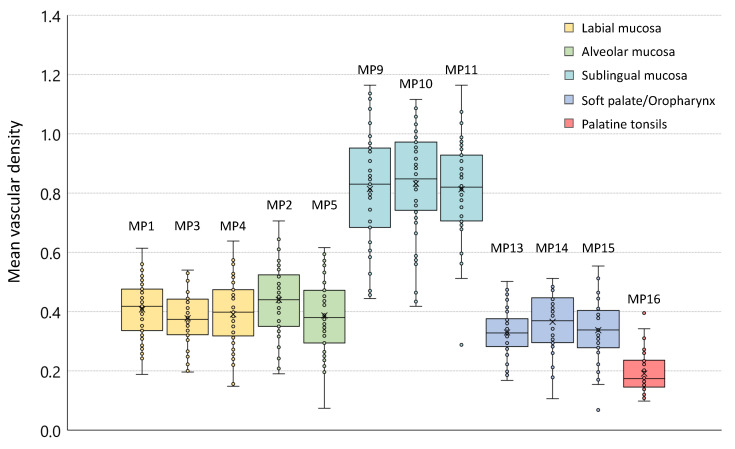
Box plot of the mean vascular density. Due to the limited penetration depth of the probing light within the buccal region and the hard palate, resulting in the limited visualization of the lamina propria and embedded vessels, reference values for the vascularization by major vessels of both regions could not be provided.

The epithelial thickness at each measurement point is outlined by means of box plots in Figure 5 providing information about the age- and sex-independent mean value, the sample median and the interquartile range. The coloration of the boxes corresponds to the investigation areas according to the measurement plan (cp. Figure 1). Focusing on the grade of tissue vascularization by means of the calculated mean vessel density (see Section 2.4), a graphical representation is realized by box plots in Figure 6.

### 3.1. Labial and Alveolar Mucosa

**Figure 7:**
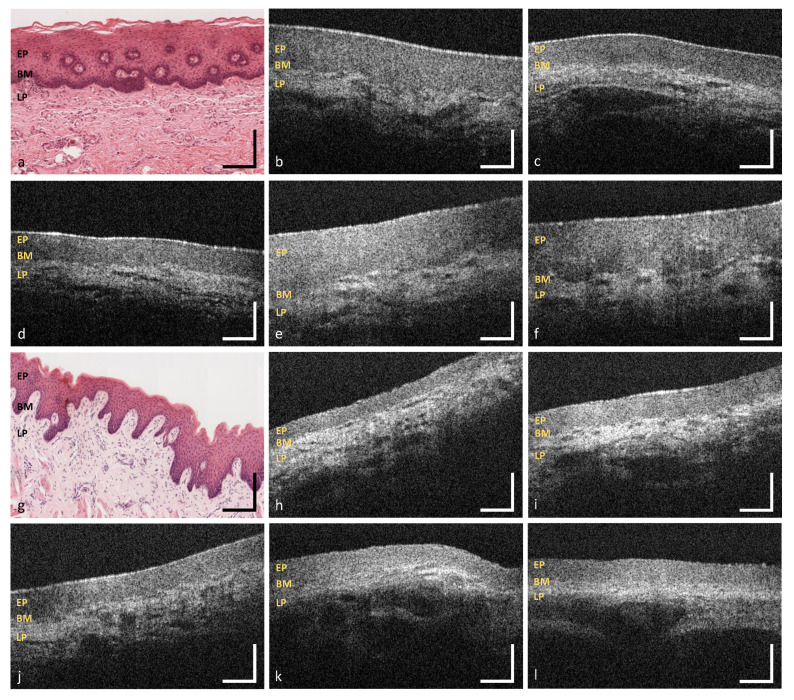
OCT images of the labial mucosa (**b**–**f**) and the alveolar mucosa (**h**–**l**). The sample pictures represent the upper (MP1) (**b**,**c**) and the lower lip (MP3,MP4) (**d**–**f**) as well as the upper (MP2) (**h**,**i**) and lower alveolar region (MP5) (**j**–**l**). Exemplary HE stained histological cross sections depicting the labial and the alveolar mucosa (**a**,**g**) ([44] modified). EP: epithelium, BM: basement membrane, LP: lamina propria. Scale bars: 200 μm.

With regard to the surface integrity and profile, labial mucosa has been evaluated as intact in 96.4% and even in 98.0% of the OCT cross sections (Figure 7b–f, Figure 4). The epithelial layer has been classified as homogeneous (98.5%) correlating to a regular epithelial structure in accordance with exemplary histological images. The total value of the labial mucosa was measured to be 243 ± 25 μm (Table A1). The basement membrane has been assessed as intact in all OCT cross sections and histological slides (e.g., Figure 7a), respectively (Figure 4). Tissue vascularization was mainly high (60.3%) with a mean ratio of 0.39±0.10 (Figure 6) due to an existing network of larger vessels [45,46]. Looking at the additional components, minor salivary glands occurred within the lamina propria in 34.5%. Although, these glands are spread over the whole oral cavity, the highest density is found within the labial, buccal, palatal and sublingual areas [42,47].

Summarizing the results of two measurement points of the alveolar region (MP2 und MP5), epithelial surface of the alveolar mucosa has been defined as intact in 92.2% and even in 95.4% (Figure 7h–i, Figure 4). The epithelial layer has been classified as homogeneous in 96.6%. The mean thickness was 142 ± 15 μm (Table A1) with no deviation between the upper and the lower alveolar area. A continuous decrease from the labial to the alveolar mucosa is already well described by histology [46] and previous OCT studies [27]. An intact basement membrane was noticed in all OCT images confirmed by exemplary histological slides (e.g., Figure 7g). The grade of tissue vascularization by means of major vessels was classified as high in 62.1% of the detected OCT cross sections with a mean ratio of 0.41±0.13 (Figure 6) being highly correlated to the mean vessel density in the labial mucosa. Moreover, clusters of salivary glands were observed in 47.4% ensuring the moistening of the alveolar region as described in histology [46].

### 3.2. Buccal Mucosa

**Figure 8:**
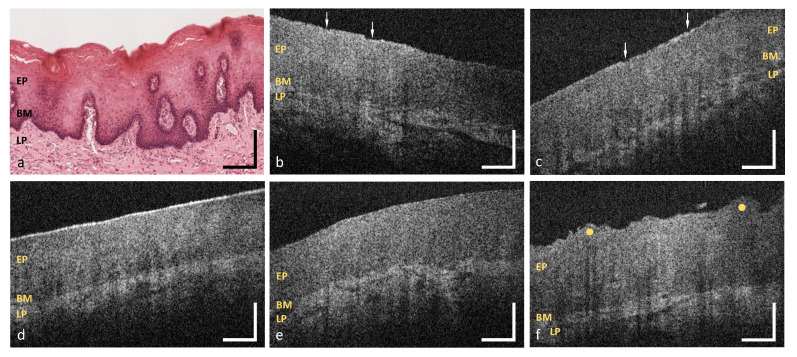
OCT images of the buccal mucosa (**b**–**f**). The sample pictures represent the anterior (MP6) (**b**,**c**), the central (MP7) (**d**) and the posterior buccal region (MP8) (**e**,**f**). Exemplary HE stained histological cross sections depicting the buccal mucosa (**a**) ([44] modified). EP: epithelium, BM: basement membrane, LP: lamina propria; Arrows: epithelial surface alteration, Yellow dots: uneven surface profile. Scale bars: 200 μm.

OCT image analysis of the buccal mucosa (Figure 8b–f) was realized by means of three measurement points depicting the anterior, the central and the posterior part. At these points, 30.6% of the OCT cross sections (Figure 8b,c arrows) showed a surface with alterations while surface profile was categorized as uneven due to a raised surface in about 14.2% (Figure 8f yellow dots). In 74.6%, the epithelium was classified as homogeneous with a mean thickness of 336±25 μm (Figure 5), being slightly thicker among the male volunteers (Table A1: MP6-8, Figure A2). Comparing the measurement points of the buccal side, epithelium was thickest at the central part. The basement membrane was intact in 60.8%, however, an amount of 22.4% was stated as not assessable due to the increased epithelial thickness and the limited imaging depth in biological tissue [36]. With regard to future studies, automated segmentation of the epithelium of the buccal mucosa could be challenging due to hyperkeratinization and a reduced visibility of the basement membrane. The grade of vascular supply by larger vessels was moderate (67.7%) with a mean ratio of 0.21±0.14 (Figure 6). Further characteristic features were not able to be detected as salivary glands are beyond the imaging depth and surrounded by fat tissue in the submucosa [46].

### 3.3. Sublingual Mucosa

**Figure 9:**
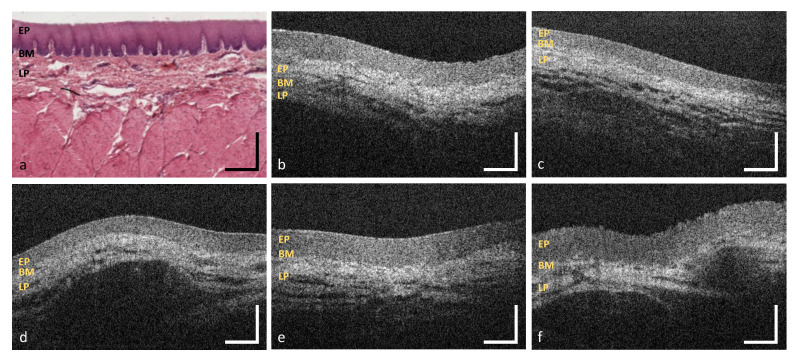
OCT images of the ventral tongue (**b**,**c**) and the mouth floor (MP11) (**d**–**f**). The sample pictures represent the anterior (MP9) (**b**) and posterior sublingual region (MP10) (**c**). Exemplary HE stained histological cross sections depicting the sublingual mucosa (**a**) ([44] modified). EP: epithelium, BM: basement membrane, LP: lamina propria. Scale bars: 200 μm.

**Figure A1:**
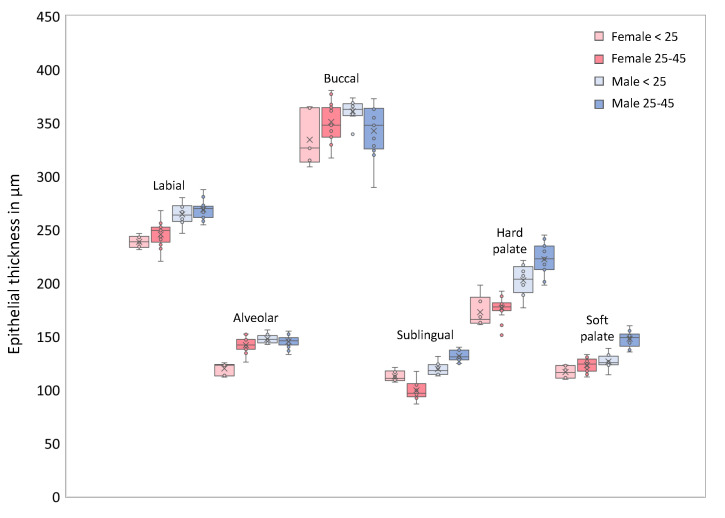
Box plot of the gender- and age-specific epithelial thickness of selected distinctive measurement points. Labial: MP4, Alveolar: MP5, Buccal: MP7, Sublingual: MP9, Hard palate: MP12, Soft palate: MP13 (cp. Figure 1).

**Figure A2:**
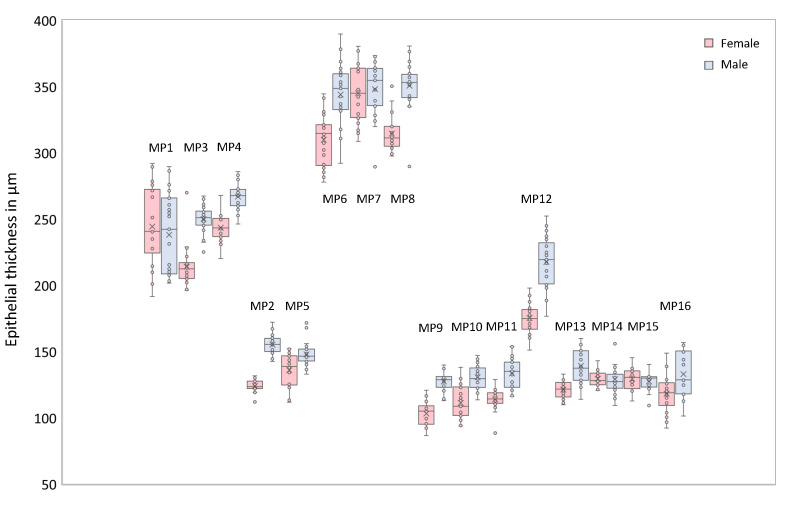
Box plot of the gender-specific epithelial thickness according to the location. Labial: MP1/MP3/MP4, Alveolar: MP2/MP5, Buccal: MP6-MP8, Sublingual: MP9/MP10, Mouth floor: MP11, Hard palate: MP12, Soft palate: MP13, Uvula: MP14, Oropharynx: MP15, Tonsils: MP16 (cp. Figure 1).

Regarding the sublingual mucosa, OCT images (Figure 9b–f) were taken from the ventral tongue (MP9, MP10) and the neighboring mouth floor (MP11), respectively. The epithelial surface appeared intact (98.9%) and even (98.2%) in agreement with all of the histological slides (e.g., Figure 9a). The epithelial structure was classified as homogeneous in 98.3% of all cases with a mean thickness of 120±15 μm (Table A1). Statistical analysis revealed significantly higher values in male participants (Table A1: MP9-11, Figure A1 and Figure A2). There were no unsharp stages determined in the course of the basement membrane. Compared to the other measurement points, the most extensive grade of vascular supply was found, because of the large sublingual arteries and veins within the lamina propria in this oral region [46]. In the present study, 86.6% of the OCT sections were evaluated as highly vascularized with a mean ratio of 0.82±0.17 (Figure 6). Beyond this, OCT images did not show any supplementary local features.

### 3.4. Hard Palate

**Figure 10:**
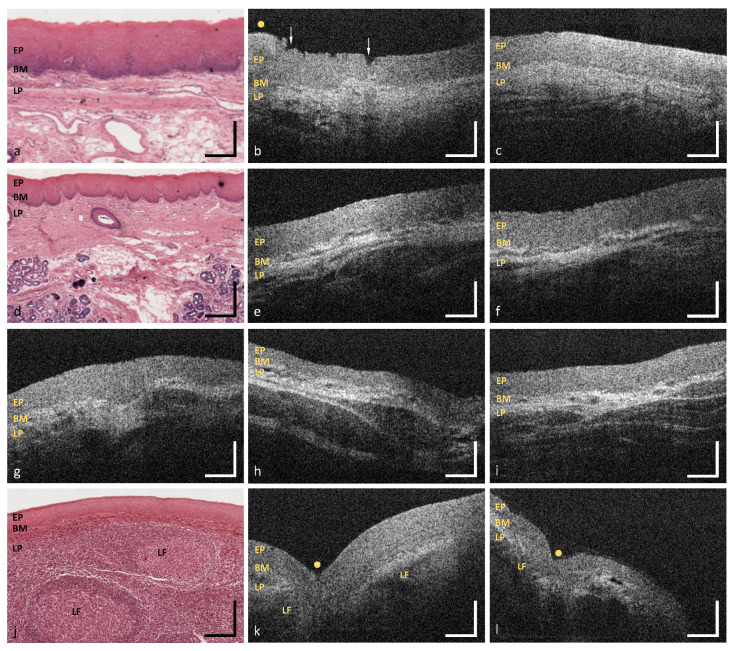
OCT images of the hard palate (MP12) (**b**,**c**), the soft palate (MP13) (**e,f**), the uvula (MP14) (**g**), the oropharynx (MP15) (**h**,**i**) and the palatine tonsil (MP16) (**k**,**l**). Exemplary HE stained histological cross sections depicting the hard palate (**a**), the soft palate (**d**) and the palatine tonsil (**j**) ([44] modified). EP: epithelium, BM: basement membrane, LP: lamina propria, LF: lymphoid follicle; Arrows: epithelial alteration, Yellow dots: palatal ridges and tonsillar crypts. Scale bars: 200 μm.

As exemplarily illustrated in (Figure 10b), in 44.6% of the detected OCT cross sections of the hard palate, multiple alterations of the epithelial surface have been detected. Moreover, the surface profile contained single or groups of convex ridges (38.8%) matching with the transverse palatal folds, as characteristic regional feature [38], which can be more or less pronounced for the selected measurement region (MP12 in Figure 1). Since hyperreflective areas have been detected below the folds, indicating a denser tissue, more than one third of the epithelial layer of the hard palate (37.5%) were classified as inhomogeneous (Figure 4). Mean epithelial thickness was 198±26 μm (Table A1) being up to 40% higher inside the convex areas. Altogether, these findings represent a physiological adaptation to the masticatory function [46]. The mean value among female volunteers (175±11 μm) was lower compared to the male (218±19 μm) (Table A1: MP12, Figure A1 and Figure A2) in accordance with former histological studies [48]. In about 34.5% of all cases, the basement membrane was assessed as unsharp due to a reduced contrast of the hyperreflective epithelial layer compared to the subsequent lamina propria. Previous studies discussed this aspect as a consequence of the keratinized surface [27]. Minor salivary glands appeared as additional components in 15.0% of the OCT cross sections, which is confirmed by histology [46].

### 3.5. Soft Palate and Oropharynx

Combining the results alongside the soft palate (MP13), the uvula (MP14) and the oropharynx (MP15), the epithelial surface was intact (96.7%) and even (96.4%), as depicted in the OCT images (Figure 10e–i) and the epithelium appeared homogeneous (89.6%). Among the measurement points, there were no significant differences in the epithelial thickness and the mean value resulted in 130±11 μm (Table A1: MP13-15, Figure A2). The basement membrane was visible and evaluated as intact in all of the OCT cross sections and the histological slides (e.g., Figure 10d), respectively. Vascularization by major vessels was rated as high in the majority of cases (56.1%) with a mean ratio of 0.34±0.09 due to a distinct vessel network in the reticular layer of the lamina propria [46]. Moreover, packets of minor salivary glands, surrounded by connective tissue, occurred in 61.1% of all cases. This feature has been also seen in histological images and described in the literature [47].

### 3.6. Palatine Tonsils

Image analysis of the palatine tonsil was performed by using OCT cross sections (Figure 10k,l) by means of a single measurement point (MP16 in Figure 1). In 76.1% of all cases, epithelial surface was categorized as intact in correspondence to the histological slides (e.g., Figure 10j). The surface profile was classified as uneven (87.5%) with concave pits that are equal to the tonsillar crypts [43]. A homogeneous (70.6%) epithelium was found with a mean thickness of 125±17 μm (Table A1) being similar to the neighboring soft palate. Looking at age-related changes, thickness was lower in the participants between 25 and 45 years compared to younger volunteers (Table A1). Confirming this aspect, an age-dependent decrease in the parenchymal lymphatic tissue combined with an increase in tissue fibrosis and fatty degeneration was reported by former histological investigations [49]. Due to a lower contrast between the epithelium and the lamina propria, the basement membrane was evaluated as unsharp in 52.2%. This finding is caused by a more permeable epithelial junction resulting in huge numbers of lymphocytes between the regularly arranged epithelial cells and in the underlying connective tissue to ensure a sufficient defense of exogenous infections [50]. In 60.3% of all cases, a moderate vascular supply was determined with a mean ratio of 0.19±0.07 (Figure 6). Regarding additional characteristic features, 86.4% of the OCT images and all histological slides showed groups of lymphoid follicles within the lamina propria as part of the primary immune function [43].

## 4. Discussion

With reference to the limited number of freely accessible OCT images concerning the healthy human oral mucosa, this study aimed to examine a representative sample of disease-free volunteers by means of an endoscopic OCT system. Looking at the study population (Table 2), the distribution between both sexes was balanced, especially within the age groups. Moreover, most participants were younger than 45 years and none of them exceeded the tolerance levels of smoking and alcohol intake or possessed visually suspicious lesions. Thus, oral soft tissue among the study population can be stated as healthy while leaving out invasive biopsies. By using the endoscopic OCT system, all of the measurement points of the oral cavity could be reached. Although some volunteers reported an uncomfortable feeling during the examination process for measurement points within the posterior oral cavity and the oropharynx, at least 60% of the cases have been accessible and therefore image data were sufficient for a representative analysis. Previous studies concerning healthy oral soft tissue concentrated on the imaging of the anterior oral mucosa [17,25,26,27], so detailed information about the accessibility of the posterior areas have not been reported before. However, the presented data suggest that this aspect should be considered for the determination of the sample size.

**Figure 11:**
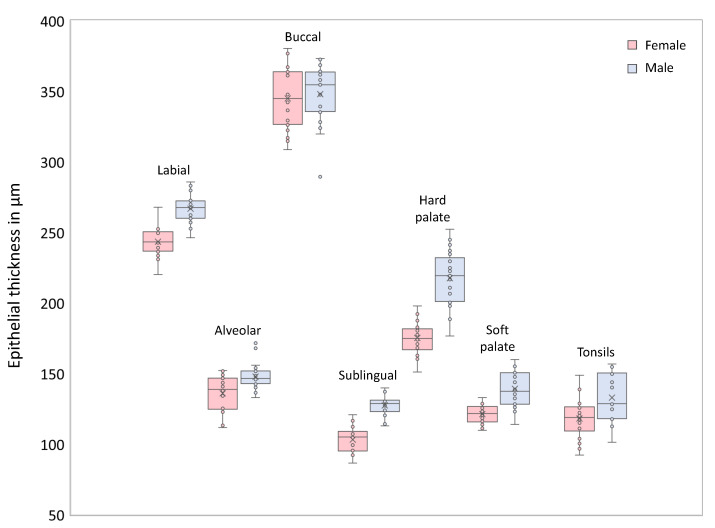
Box plot of the gender-specific epithelial thickness of recommended measurement points. The limited selection of distinctive measurement points is conceivable to serve as prospective parameter for assessing oral health. Labial: MP4, Alveolar: MP5, Buccal: MP7, Sublingual: MP9, Hard palate: MP12, Soft palate: MP13, Tonsils: MP16.

Regarding the results of the epithelial thickness measurement, the lowest mean values were determined for the sublingual mucosa (120±15 μm) in accordance with the reference values of the mouth floor (100±14 μm) by Stasio et al. [27]. Additionally, Stasio et al. provided comparable results for the labial mucosa (271±36 μm) and Prestin et al. [25] presented similar data for the anterior mouth floor (99±22 μm), the uvula (144±30 μm) and the hard palate (239±57 μm). The highest mean value within the presented study was calculated for the buccal mucosa with about 336±25 μm. At this point, Stasio et al. reported a mean value of 374±62 μm agreeing with the reference value of Prestin et al. (294±68 μm). One should consider, that the results for the buccal mucosa by Prestin et al. were received by measurements with direct contact containing the influence of tissue stretching. In this study, measurement process was performed contact-free for which reason local tissue compression could be avoided. On the other hand, the penetration depth of the probing light was limited by a thick epithelium reducing the visibility of the basement membrane and the subsequent lamina propria. Thus, the thickness measurement of the buccal epithelium by the presented EOCT system is limited. Histological investigations reported a mean value of 480±90 μm for the buccal mucosa [51]. Deviations between in vivo OCT and histology could be reasoned in tissue fixation and histological preparation of the physiological oral mucosa [52]. In compliance with our expectations, the epithelial thickness of the measurement points did not show notable differences within the investigation areas (MP6-MP8), and thus, reference values can be defined by means of a reduced number of recommended measurement points as shown in the diagram (Figure 11).

The assessment of tissue vascularization was realized by calculating the mean vascular density by means of major vessels. Considering the anatomical structure of the oral mucosa, the luminal cross-sectional area of larger vessels was related to the avascular epithelium. Apart from the buccal mucosa and the hard palate where measurement was restricted due to a limited penetration depth, a moderate to high grade of vascularization was found within the defined investigation areas (Figure 1). Setting these results in a wider context, previous OCT studies used different approaches to quantify vascular supply [40,45]. Tsai et al. [40] calculated an average vessel density by using en-face projections of OCT angiographic images. At a depth of 250 to 500 μm, a high vascularization was determined for the labial mucosa, the buccal mucosa and the sublingual mucosa, respectively. For future research on pathologies, the combination of intensity-based OCT and functional imaging, e.g., Doppler OCT and OCT angiography, is recommended while concentrating on both, capillaries and large blood and lymphatic vessels.

Looking at the qualitative image criteria, the evaluation of the OCT cross sections provided similar results for the labial mucosa, the alveolar mucosa, the sublingual mucosa and the soft palate, respectively. There, OCT cross sections displayed an even and homogeneous epithelial layer without surface alterations that is clearly demarcated by a visually consistent basement membrane. With reference to previous histological descriptions [46], these findings correlate with the anatomical structure of the lining mucosa, which includes an intact and regular epithelium with a smooth border to the adjacent lamina propria. The appearance of lining mucosa in OCT was analyzed by Ridgway et al. [26] using sample pictures of the lower lip, the ventral side of the tongue and the mouth floor showing a homogeneous hyporeflective epithelium and a hyperreflective lamina propria. Gentile et al. [17] presented a similar characterization in the context of a systematic review as reference for future investigations. However, epithelial surface has not been widely examined by means of OCT before due to the absence of established criteria. Although the buccal mucosa is histologically classified as lining mucosa as well, in vivo OCT images showed an epithelium containing numerous alterations and inhomogeneous areas. According to former histologic reports [46], this feature can be explained as a consequence of mechanical stress leading to epithelial damage and local reactive keratinization. Concerning the epithelial homogeneity, Stasio et al. [27] described regional changes in the epithelial layer as a result of surface keratinization. OCT cross sections of the hard palate displayed an epithelium with multiple alterations and a keratinized surface as typical feature of the masticatory mucosa in agreement with histological examinations [46]. For the first time, the presented study provided detailed information about the surface profile by delivering in vivo OCT pictures of the transverse palatal folds.

Concerning the depiction of minor salivary glands, the used OCT system allowed the identification of clusters that are located close to the basement membrane [53]. The appearance in the OCT images was accordant with the description of Grulkowski et al. [42] in the form of round hyporeflective elements with an excretory duct surrounded by connective tissue. However, for the depiction of deeper-lying clusters of minor salivary glands, the usage of an OCT system with a center wavelength of 1300 nm is recommended.

## 5. Summary and Conclusions

In the presented clinical study, in vivo OCT cross sections of the healthy human oral mucosa were obtained with the aim of generating a standardized image analysis of intensity-based OCT cross sections. Concentrating on cancer-relevant parts of the oral cavity and the oropharyngeal isthmus, a total of 47 volunteers was examined gathering more than 3500 OCT images out of seven investigation areas by means of an EOCT system. In extension to former OCT studies, information about the not readily accessible posterior areas, such as the oropharynx and the palatine tonsil, was achieved. Concerning the image evaluation, a list of seven criteria was implemented especially considering the integrity and the profile of the epithelial surface as well as the grade of vascular supply by major vessels as future indicators for the detection of suspicious oral lesions. Moreover, the extent of freely available OCT cross sections was enlarged by creating a collection of sample pictures demonstrating regional characteristics to expand the current state of knowledge about the healthy adult oral soft tissue. In order to prevent unnecessary biopsies, histological correlation was realized using HE stained image material out of a virtual database of human tissue. Altogether, there was a good correlation between the OCT cross sections and the histological slides as diagnostic gold standard.

To the best of our knowledge, for the first time mean values for the epithelial thickness of the alveolar mucosa, the palatine tonsil and the oropharynx were obtained from OCT images. Comparing the results of different investigation areas, epithelium of the sublingual mucosa was found to be thinnest in contrast to a maximum alongside the buccal region. Furthermore, the influence of sex and age on the epithelium was examined by dividing the sample into groups. Within the sublingual and the palatal region, mean epithelial thickness was increased in male subjects while age-related variations were found at the palatine tonsil. According to the anatomical configuration, mean vascular density was measured in relation to the avascular epithelium. Focusing on major vessels, a moderate to high tissue vascularization was determined within the observed regions including a maximum alongside the sublingual area. In a wider context, the assessment might be extended by quantifying the number of capillaries by means of imaging techniques like OCT angiography.

Concerning the analysis of the qualitative criteria, areas of lining mucosa appeared similar in the OCT cross sections possessing an even and homogeneous epithelium without alterations followed by a visually intact basement membrane. Within the buccal region, the hard palate and the palatine tonsil, respectively, oral mucosa showed local features representing physiological adaptations to mechanical stress or immune functions. Altogether, the findings suggest that an appropriate characterization of the healthy oral soft tissue can be achieved with a few measurement points.

Looking at disease-free individuals without alcohol or tobacco consumption above the tolerance levels, this study also indicates fields of further research. As an example, visually normal oral soft tissue in subjects with increased alcohol and tobacco intake need to be investigated for reaching a better understanding of early changes. Beyond that, the results should be the basis for future clinical studies aiming to improve the differentiation of suspicious lesions into benign and malignant.

With regard to the examination process, image analysis confirmed that future in vivo measurements can be simplified by gathering short series of OCT cross sections from a limited number of sites inside the investigation areas. In addition, there is a potential for the automatic epithelial thickness measurement based on the presented data in combination with future studies with an increased number of image data sets and analysts. In clinical practice, OCT might serve as adjunctive screening tool that is used to complement the visual evaluation and to facilitate the determination of the biopsy site. Against this background, the presented study offers a recommendation for the selection of the measurement points and comprehensive data about the expected appearance in OCT images.

## Figures and Tables

**Table 1:** Criteria of the OCT image analysis.

Criterion	Evaluation
Surface integrity	intact/with alterations
Surface profile	even/uneven
Epithelial homogeneity	homogeneous/inhomogeneous
Epithelial thickness	mean value of 25 measurements (μm)
Basement membrane	intact/unsharp/not assessable
Tissue vascularization	low (<0.10)/moderate (0.10–0.30)/high (>0.30)
Additional components	Description of regional features within the lamina propria

**Table 2:** Statistics on the study population and the amount of the OCT cross sections.

Study Population		OCT Cross Sections	
Nb. of volunteers	47	Investigation area	Nb. of images
Male	25 (53.2%)
Female	22 (46.8%)	Labial mucosa	705
Mean age (years)	29.36	Alveolar mucosa	470
<25 years	14 (29.8%)	Buccal mucosa	705
25–45 years	30 (63.8%)	Sublingual mucosa	705
>45 years	3 (6.4%)	Hard palate	235
Oral symptoms/lesions	0	Soft palate & oropharynx	570
Alcohol intake > TL ^1^	0	Palatine tonsils	170
Never drinking	19 (40.4%)
Tobacco consumption > TL ^1^	0	*Total*	3,560
Never smoking	20 (42.6%)		

^1^ Tolerance level.

**Figure A3:**
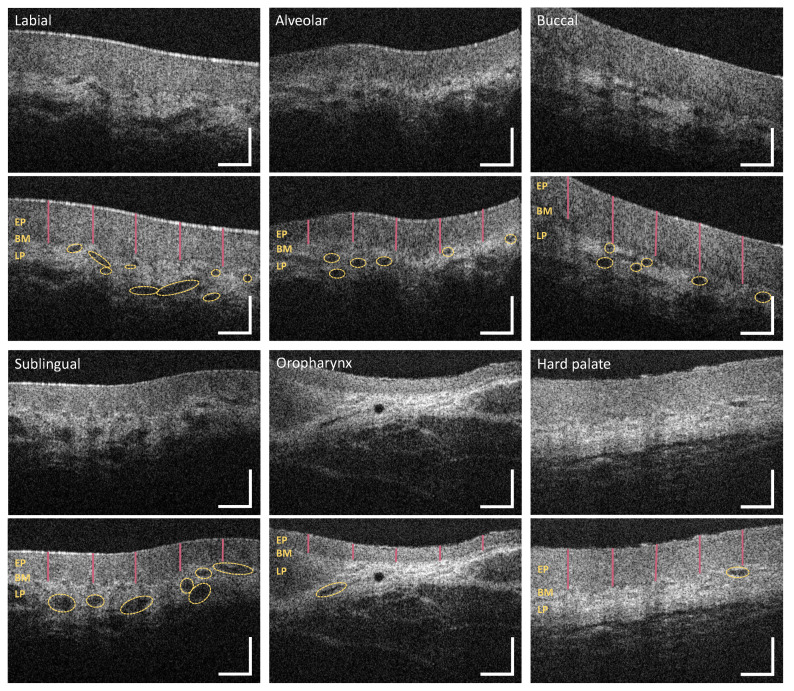
Representative OCT images of the human oral mucosa in vivo of the labial, alveolar, buccal and sublingual region as well as the oropharynx and the hard palate demonstrating the principle of the visual image analysis. EP: epithelium, BM: basement membrane, LP: lamina propria; Vertical lines: epithelial thickness measurement, Ellipsoidal shapes: marked regions of vascularization. Scale bars: 200 μm.

**Figure A4:**
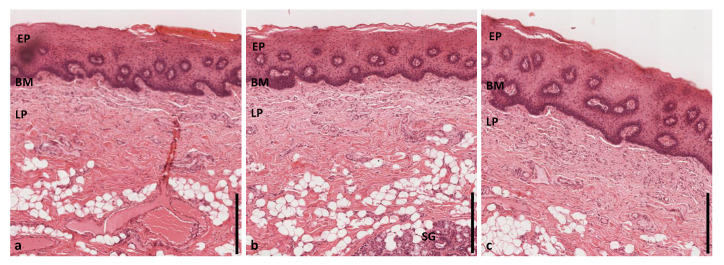
Supplementary HE stained histological cross sections of the human labial oral mucosa [44]. EP: epithelium, BM: basement membrane, LP: lamina propria, SG: salivary gland. Scale bar: 200 μm.

**Figure A5:**
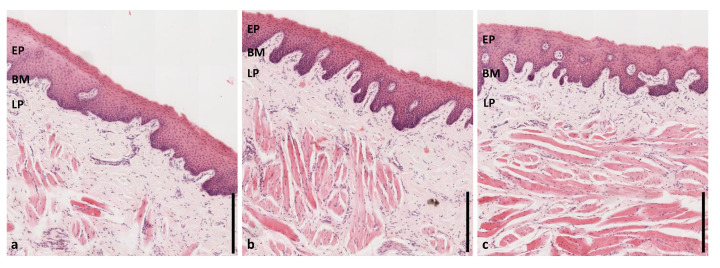
Supplementary HE stained histological cross sections of the human alveolar mucosa [44]. EP: epithelium, BM: basement membrane, LP: lamina propria, Scale bar: 200 μm.

**Figure A6:**
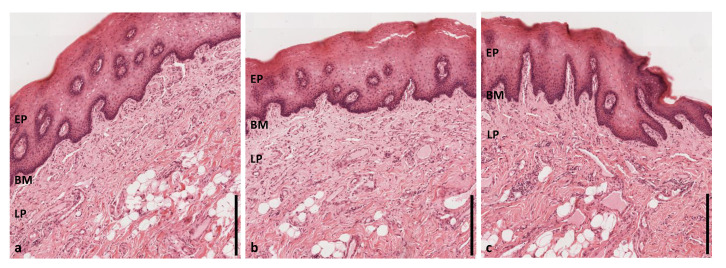
Supplementary HE stained histological cross sections of the human buccal mucosa [44]. EP: epithelium, BM: basement membrane, LP: lamina propria, Scale bar: 200 μm.

**Figure A7:**
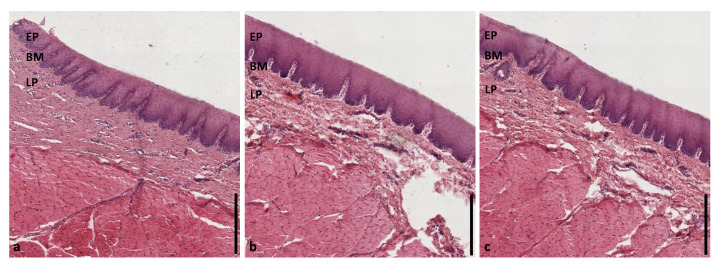
Supplementary HE stained histological cross sections of the human sublingual mucosa [44]. EP: epithelium, BM: basement membrane, LP: lamina propria, Scale bar: 200 μm.

**Figure A8:**
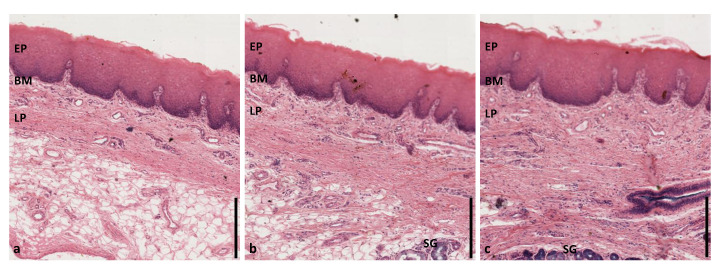
Supplementary HE stained histological cross sections of the mucosal tissue of the human hard palate [44]. EP: epithelium, BM: basement membrane, LP: lamina propria, SG: salivary gland. Scale bar: 200 μm.

**Figure A9:**
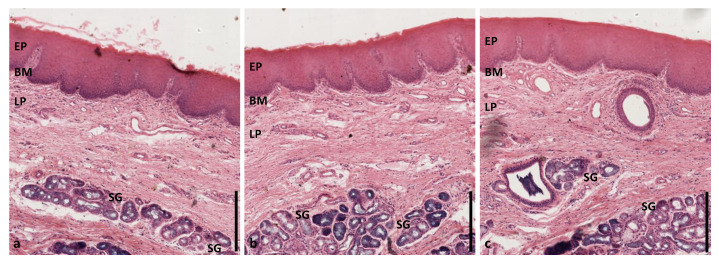
Supplementary HE stained histological cross sections of the mucosal tissue of the human soft palate [44]. EP: epithelium, BM: basement membrane, LP: lamina propria, SG: salivary gland. Scale bar: 200 μm.

**Figure A10:**
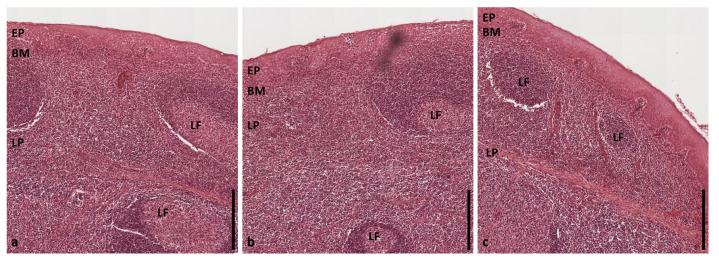
Supplementary HE stained histological cross sections of the mucosal tissue of the human tonsil [44]. EP: epithelium, BM: basement membrane, LP: lamina propria, LF: lymphoid follicle. Scale bar: 200 μm.

**Figure A11:**
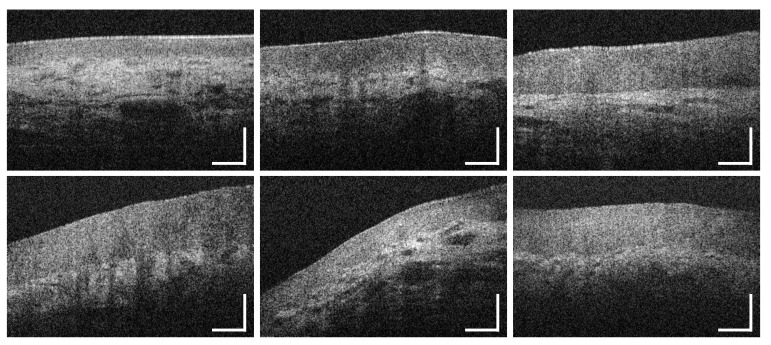
Supplementary intensity-based OCT cross sections of the mucosal tissue of the inner upper lip (MP1, Figure 1). Scale bars: 200 μm.

**Figure A12:**
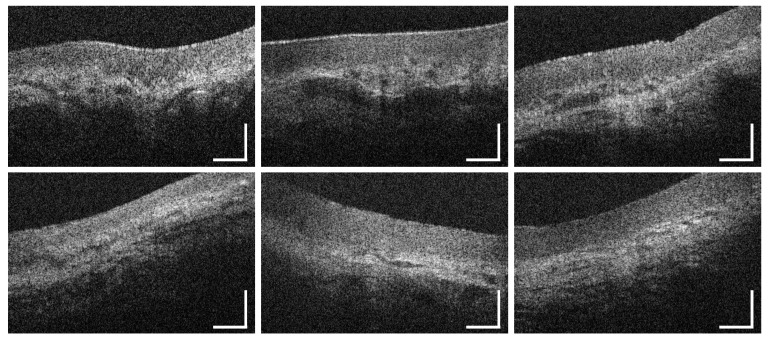
Supplementary intensity-based OCT cross sections of the alveolar mucosa of the upper vestibular region (MP2, Figure 1). Scale bars: 200 μm.

**Figure A13:**
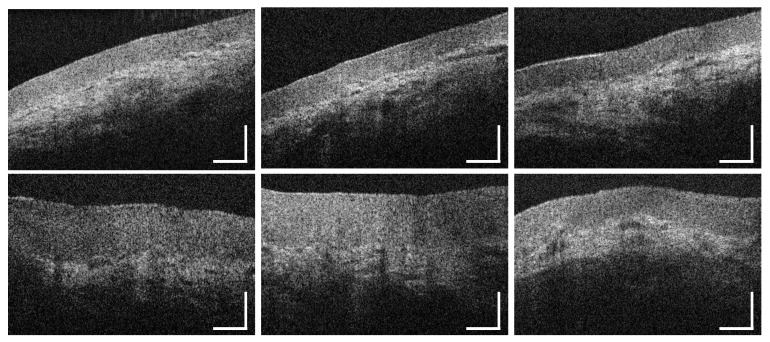
Supplementary intensity-based OCT cross sections of the mucosal tissue of the central inner lower lip (MP3, Figure 1). Scale bars: 200 μm.

**Figure A14:**
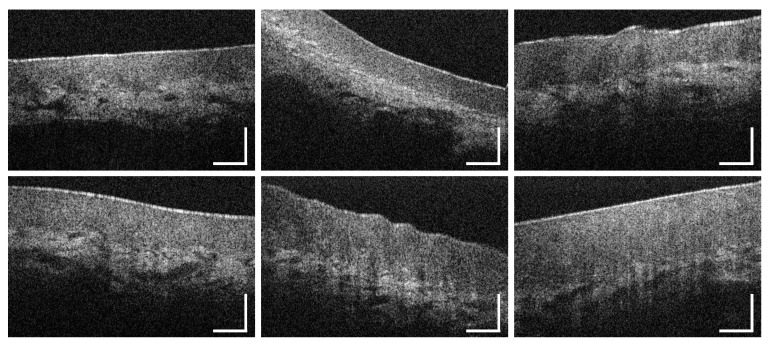
Supplementary intensity-based OCT cross sections of the mucosal tissue of the lateral inner lower lip (MP4, Figure 1). Scale bars: 200 μm.

**Figure A15:**
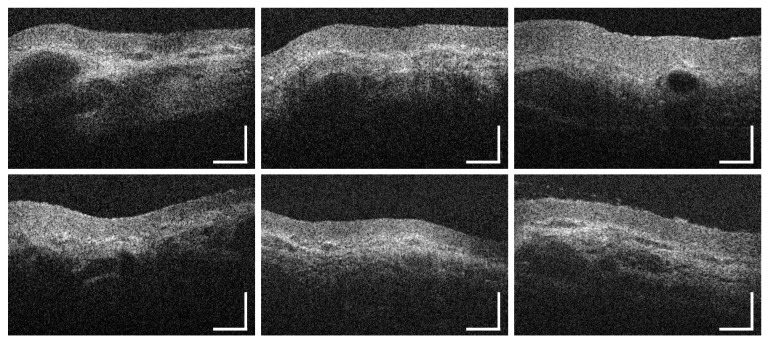
Supplementary intensity-based OCT cross sections of the alveolar mucosa of the lower vestibular region (MP5, Figure 1). Scale bars: 200 μm.

**Figure A16:**
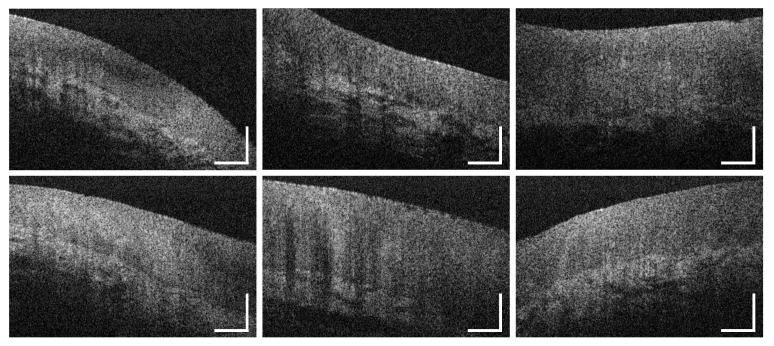
Supplementary intensity-based OCT cross sections of the buccal mucosa of the anterior inner cheek (MP6, Figure 1). Scale bars: 200 μm.

**Figure A17:**
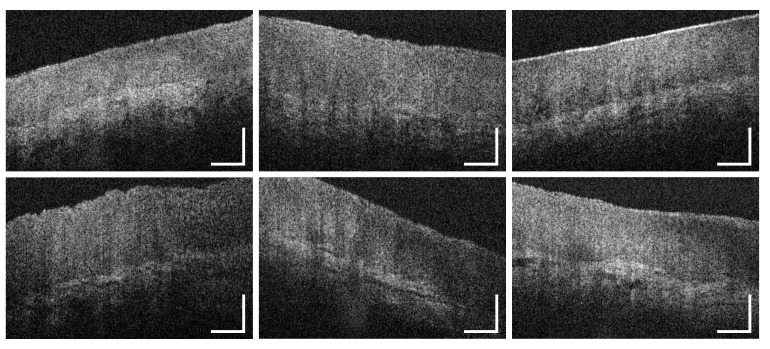
Supplementary intensity-based OCT cross sections of the buccal mucosa of the central inner cheek (MP7, Figure 1). Scale bars: 200 μm.

**Figure A18:**
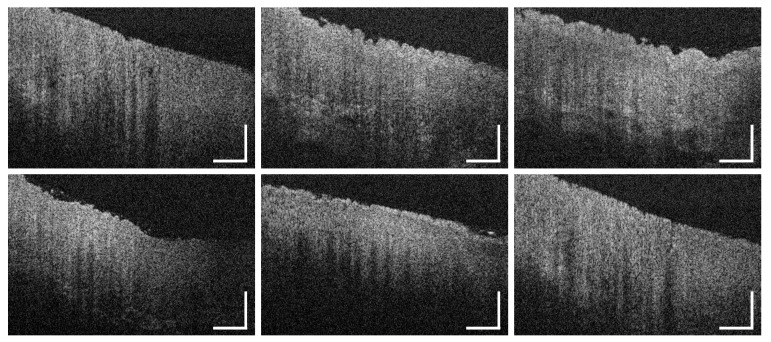
Supplementary intensity-based OCT cross sections of the buccal mucosa of the posterior inner cheek (MP8, Figure 1). Scale bars: 200 μm.

**Figure A19:**
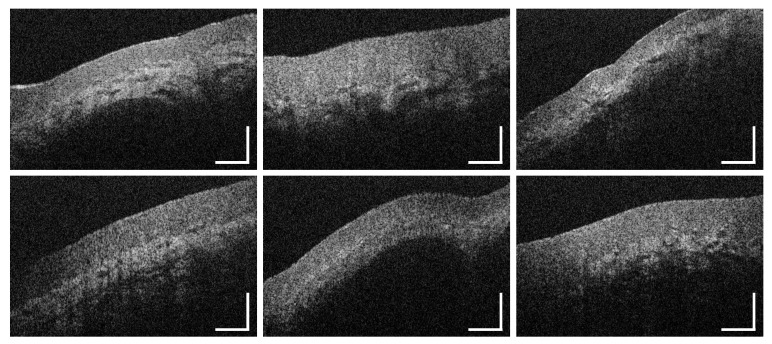
Supplementary intensity-based OCT cross sections of the oral mucosa of the anterior ventral tongue (MP9, Figure 1). Scale bars: 200 μm.

**Figure A20:**
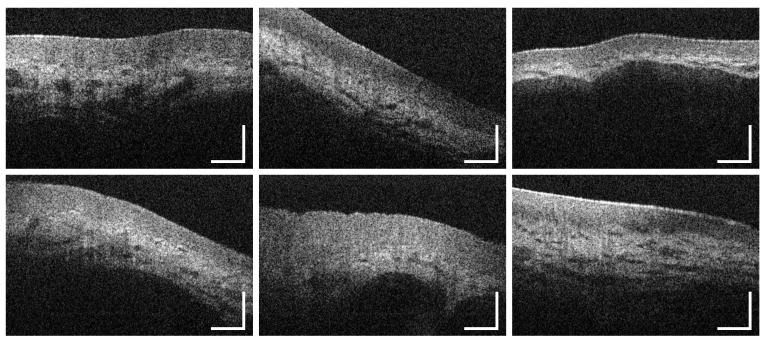
Supplementary intensity-based OCT cross sections of the oral mucosa of the central ventral tongue (MP10, Figure 1). Scale bars: 200 μm.

**Figure A21:**
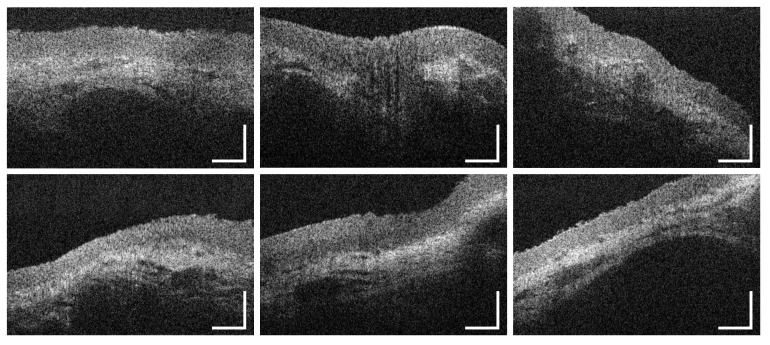
Supplementary intensity-based OCT cross sections of the mucosal tissue of the mouth floor (MP11, Figure 1). Scale bars: 200 μm.

**Figure A22:**
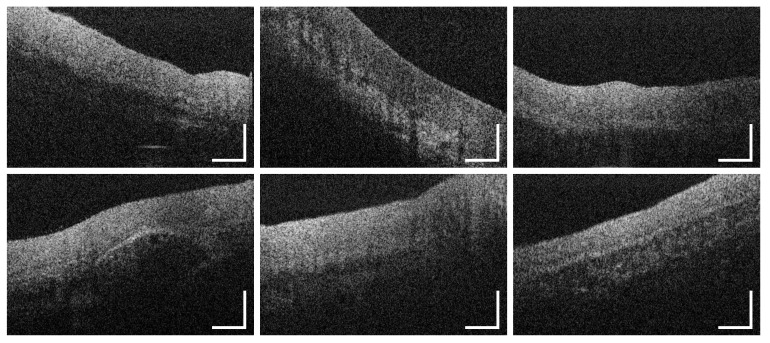
Supplementary intensity-based OCT cross sections of the oral muucosa of the hard palate (MP12, Figure 1). Scale bars: 200 μm.

**Figure A23:**
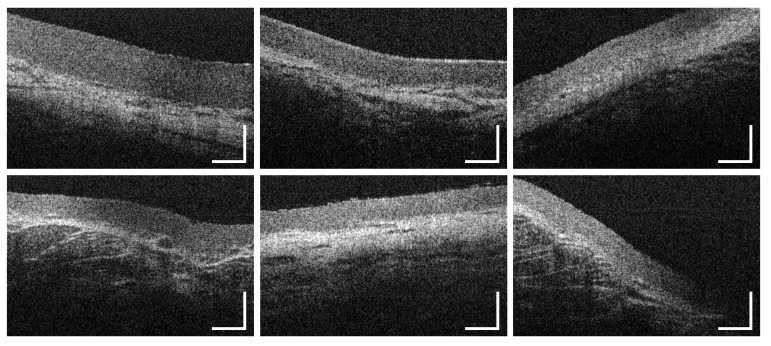
Supplementary intensity-based OCT cross sections of the mucosal tissue of the soft palate (MP13, Figure 1). Scale bars: 200 μm.

**Figure A24:**
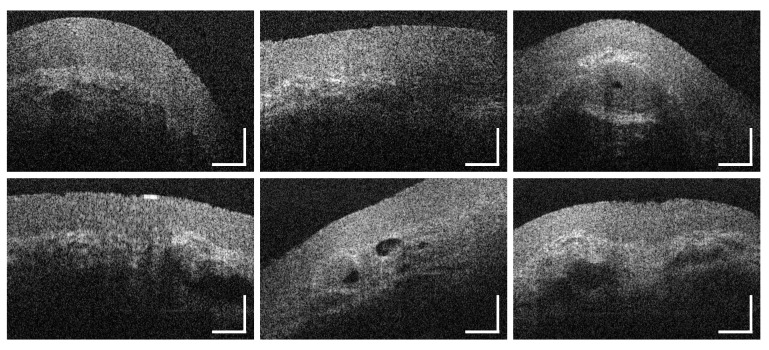
Supplementary intensity-based OCT cross sections of the oral mucosa of the uvula (MP14, Figure 1). Scale bars: 200 μm.

**Figure A25:**
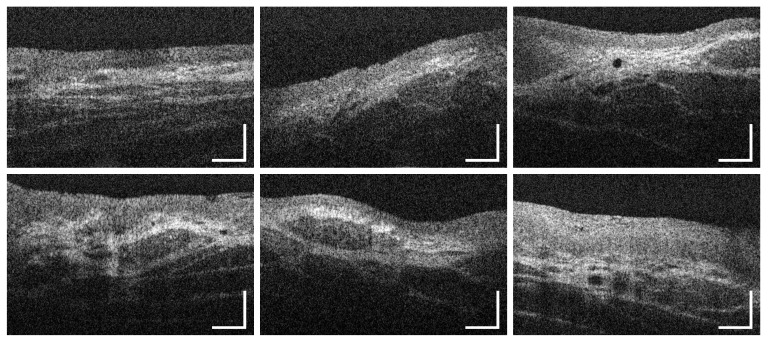
Supplementary intensity-based OCT cross sections of the mucosal tissue of the oropharynx (MP15, Figure 1). Scale bars: 200 μm.

**Figure A26:**
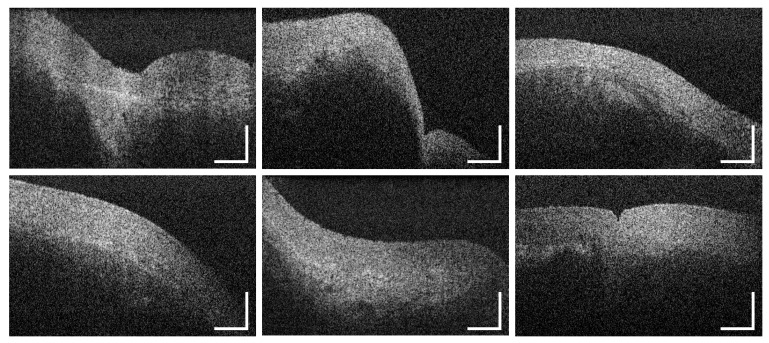
Supplementary intensity-based OCT cross sections of the mucosal tissue of the palatine tonsil (MP16, Figure 1). Scale bars: 200 μm.

## Appendix A

**Table A1:** Mean epithelial thickness (± standard deviation) of individual measurement points and defined regions adjusted by sex and age.

Measurement Point/Region	Sex Distribution		Age Group			Total
	Male	Female	< 25	25–45	> 45	
MP 1	238±30μm	244±30μm	250±32μm	236±28μm	250±37μm	241±30μm
MP 2	155±7μm	124±5μm	140±15μm	140±17μm	147±31μm	141±17μm
MP 3	250±10μm	214±15μm	232±18μm	231±23μm	264±7μm	233±22μm
MP 4	267±9μm	244±10μm	253±16μm	258±15μm	253±9μm	256±15μm
MP 5	148±9μm	136±12μm	136±15μm	144±7μm	162±13μm	142±12μm
MP 6	344±23μm	310±19μm	327±34μm	327±24μm	341±18μm	328±27μm
MP 7	348±20μm	345±21μm	349±22μm	347±21μm	333±10μm	347±21μm
MP 8	351±18μm	315±13μm	336±24μm	333±24μm	331±28μm	334±24μm
MP 9	128±8μm	103±9μm	116±7μm	116±18μm	121±14μm	116±15μm
MP 10	131±9μm	110±12μm	124±7μm	120±17μm	119±14μm	121±15μm
MP 11	134±11μm	114±8μm	121±5μm	127±17μm	117±5μm	124±14μm
MP 12	218±19μm	175±11μm	190±21μm	199±26μm	220±43μm	198±26μm
MP 13	139±12μm	121±7μm	122±8μm	135±14μm	131±7μm	131±13μm
MP 14	129±12μm	129±6μm	126±7μm	129±7μm	148±14μm	129±9μm
MP 15	128±8μm	130±8μm	131±7μm	128±7μm	122±12μm	129±8μm
MP 16	133±17μm	118±14μm	142±15μm	115±11μm	141±3μm	125±17μm
Labial mucosa	252±22μm	234±24μm	245±25μm	242±25μm	256±20μm	243±25μm
Alveolar mucosa	152±9μm	130±11μm	138±15μm	142±13μm	155±23μm	142±15μm
Buccal mucosa	348±20μm	323±24μm	338±28μm	336±24μm	335±18μm	336±25μm
Sublingual mucosa	131±10μm	109±11μm	121±7μm	121±18μm	119±10μm	120±15μm
Hard palate	218±19μm	175±11μm	190±21μm	199±26μm	220±43μm	198±26μm
Soft palate and oropharynx	133±12μm	126±8μm	126±8μm	131±11μm	134±15μm	130±11μm
Palatine tonsils	133±17μm	118±14μm	142±15μm	115±11μm	141±3μm	125±17μm

**Table A2:** Mean vascular density of specific oral measurement points and regions. Due to the limited visualization of the lamina propria for the inner cheek and the hard palate, vascularization by major vessels of both regions could not be determined.

Measurement Point/Region	Vascularization
MP1	0.42±0.10
MP2	0.44±0.12
MP3	0.38±0.09
MP4	0.39±0.11
MP5	0.39±0.12
MP9	0.81±0.20
MP10	0.83±0.17
MP11	0.81±0.16
MP13	0.33±0.08
MP14	0.37±0.10
MP15	0.34±0.11
MP16	0.19±0.07
Labial mucosa	0.39±0.10
Alveolar mucosa	0.41±0.13
Sublingual mucosa	0.82±0.17
Soft palate and oropharynx	0.34±0.09
Palatine tonsils	0.19±0.07

**Table A3:** Success rate of OCT data collection per measurement point, participant and investigation area.

Participant Number	Age Group	Sex	Measurement Point																Success Rate (%)
			1	3	4	2	5	6	7	8	9	10	11	12	13	14	15	16	
1	(1)	m	x	x	x	x	x	x	x	x	x	x	x	x	x	x	x	x	100
2	(1)	m	x	x	x	x	x	x	x	x	x	x	x	x	x	x	x	x	100
3	(1)	m	x	x	x	x	x	x	x	x	x	x	x	x	x	x	x	x	100
4	(1)	m	x	x	x	x	x	x	x	x	x	x	x	x	x	x	x	x	100
5	(1)	m	x	x	x	x	x	x	x	x	x	x	x	x	x	x	0	x	93.8
6	(1)	m	x	x	x	x	x	x	x	x	x	x	x	x	x	x	0	0	87.5
7	(1)	m	x	x	x	x	x	x	x	x	x	x	x	x	x	x	0	0	87.5
8	(1)	m	x	x	x	x	x	x	x	x	x	x	x	x	x	0	0	0	81.3
9	(2)	m	x	x	x	x	x	x	x	x	x	x	x	x	x	x	x	x	100
10	(2)	m	x	x	x	x	x	x	x	x	x	x	x	x	x	x	x	x	100
11	(2)	m	x	x	x	x	x	x	x	x	x	x	x	x	x	x	x	x	100
12	(2)	m	x	x	x	x	x	x	x	x	x	x	x	x	x	x	x	x	100
13	(2)	m	x	x	x	x	x	x	x	x	x	x	x	x	x	x	x	x	100
14	(2)	m	x	x	x	x	x	x	x	x	x	x	x	x	x	x	x	x	100
15	(2)	m	x	x	x	x	x	x	x	x	x	x	x	x	x	x	x	x	100
16	(2)	m	x	x	x	x	x	x	x	x	x	x	x	x	x	x	0	x	93.8
17	(2)	m	x	x	x	x	x	x	x	x	x	x	x	x	x	x	0	x	93.8
18	(2)	m	x	x	x	x	x	x	x	x	x	x	x	x	x	x	0	0	87.5
19	(2)	m	x	x	x	x	x	x	x	x	x	x	x	x	x	x	0	0	87.5
20	(2)	m	x	x	x	x	x	x	x	x	x	x	x	x	x	x	0	0	87.5
21	(2)	m	x	x	x	x	x	x	x	x	x	x	x	x	x	0	0	0	81.3
22	(2)	m	x	x	x	x	x	x	x	x	x	x	x	x	0	0	0	0	75.0
23	(2)	m	x	x	x	x	x	x	x	x	x	x	x	x	0	0	0	0	75.0
24	(3)	m	x	x	x	x	x	x	x	x	x	x	x	x	x	x	x	x	100
25	(3)	m	x	x	x	x	x	x	x	x	x	x	x	x	x	x	x	x	100
26	(1)	f	x	x	x	x	x	x	x	x	x	x	x	x	x	x	x	x	100
27	(1)	f	x	x	x	x	x	x	x	x	x	x	x	x	x	x	x	x	100
28	(1)	f	x	x	x	x	x	x	x	x	x	x	x	x	x	x	x	x	100
29	(1)	f	x	x	x	x	x	x	x	x	x	x	x	x	x	x	0	x	93.8
30	(1)	f	x	x	x	x	x	x	x	x	x	x	x	x	x	0	0	0	81.3
31	(2)	f	x	x	x	x	x	x	x	x	x	x	x	x	x	x	x	x	100
32	(2)	f	x	x	x	x	x	x	x	x	x	x	x	x	x	x	x	x	100
33	(2)	f	x	x	x	x	x	x	x	x	x	x	x	x	x	x	x	x	100
34	(1)	f	x	x	x	x	x	x	x	x	x	x	x	x	x	x	x	x	100
35	(2)	f	x	x	x	x	x	x	x	x	x	x	x	x	x	x	x	x	100
36	(2)	f	x	x	x	x	x	x	x	x	x	x	x	x	x	x	x	x	100
37	(2)	f	x	x	x	x	x	x	x	x	x	x	x	x	x	x	x	x	100
38	(2)	f	x	x	x	x	x	x	x	x	x	x	x	x	x	x	x	x	100
39	(2)	f	x	x	x	x	x	x	x	x	x	x	x	x	x	x	x	x	100
40	(2)	f	x	x	x	x	x	x	x	x	x	x	x	x	x	x	x	x	100
41	(2)	f	x	x	x	x	x	x	x	x	x	x	x	x	x	x	x	x	100
42	(2)	f	x	x	x	x	x	x	x	x	x	x	x	x	x	x	x	x	100
43	(2)	f	x	x	x	x	x	x	x	x	x	x	x	x	x	x	0	x	93.8
44	(2)	f	x	x	x	x	x	x	x	x	x	x	x	x	x	x	0	0	87.5
45	(2)	f	x	x	x	x	x	x	x	x	x	x	x	x	x	x	0	0	87.5
46	(2)	f	x	x	x	x	x	x	x	x	x	x	x	x	x	0	0	0	100
47	(3)	f	x	x	x	x	x	x	x	x	x	x	x	x	x	x	x	x	100
**Investigation area**			**Labial mucosa**			**Alveolar mucosa**		**Buccal mucosa**			**Sublingual mucosa**			**Hard palate**	**Soft palate/oropharynx**			**Palatine tonsil**	
**Success rate %**			**100**			**100**		**100**			**100**			**100**	**95.7**	**85.1**	**61.7**	**72.3**	

Age group: (1): <25; (2): 25–45; (3): >45. Sex: m: male; f = female. Success rate: Percentage of measurement points reached (x: OCT data collected, 0: no OCT data).
